# Association between dental flossing frequency and oral microbiome in U.S. adults

**DOI:** 10.1080/07853890.2026.2614826

**Published:** 2026-01-16

**Authors:** Zhijing Xu, Jinyu Hu, Huabin Luo, Xiang Qi, Ruotong Liu, Yunrui Liu, Yaguang Zheng, Huilin Li, Bei Wu

**Affiliations:** a New York University Rory Meyers College of Nursing, New York, NY, USA; b East Carolina University Brody School of Medicine, Greenville, NC, USA; c New York University School of Medicine, New York, NY, USA; d New York University Shanghai, Shanghai, China

**Keywords:** α-diversity, β-diversity, dental hygiene, dentistry, oral microbiome, oral health

## Abstract

**Background:**

The oral microbiome is vital for health, yet population-based evidence on how self-reported flossing relates to microbial communities remains limited. This study examined the association between self-reported dental flossing frequency and oral microbiome diversity in a nationally representative sample of U.S. adults.

**Methods:**

This cross-sectional analysis included 4,772 adults aged 30-69 from NHANES 2009–2012. Flossing frequency was categorized as non-users (0 days/week), some flossing (1-6 days/week), and daily users (7 days/week). Oral microbiome composition was profiled using 16S rRNA sequencing. α-diversity was calculated using Observed amplicon sequence variants (ASVs), Shannon, Inverse Simpson, and Faith’s Phylogenetic Diversity (PD); β-diversity using Bray–Curtis and UniFrac distances. Survey-weighted linear regression and PERMANOVA were used with covariate adjustment.

**Results:**

Participants included, non-users (32%), some flossing (38%), and daily users (30%). A dose–response relationship was observed between flossing frequency and reduced microbial richness and phylogenetic diversity. Compared with non-users, daily users exhibited significantly lower richness (Observed ASVs: β = −11.46, 95% CI: −15.62 to −7.29) and phylogenetic diversity (Faith’s PD: β = −0.88, 95% CI: −1.20 to −0.56). Daily flossing was associated with a modest reduction in Shannon diversity, with no significant association for the Inverse Simpson index. Inverse associations were more pronounced among younger and lower-income adults, but not among current smokers. β-diversity differed significantly across groups, although effect sizes were minimal (Bray–Curtis *R*^2^ = 0.059%; unweighted UniFrac *R*^2^ = 0.090%).

**Conclusions:**

Frequent flossing was associated with reduced microbial richness and phylogenetic diversity, potentially indicating a favorable shift toward a healthier microbial community.

## Key Messages

1. In a nationally representative sample of U.S. adults, a higher frequency of dental flossing, particularly daily use, is associated in a dose-dependent manner with lower oral microbiome richness and phylogenetic diversity.
2. The inverse association between flossing frequency and microbial diversity is stronger among younger adults and individuals with lower socioeconomic status, but is not observed among current smokers.
3. Despite small effect sizes, flossing frequency is associated with statistically significant shifts in overall oral microbial community structure (β-diversity).

## Background

The human oral cavity harbors one of the most complex microbial ecosystems in the human body, comprising over 700 bacterial species that form dynamic communities essential to both oral and systemic health [1]. While the oral microbiome supports metabolic functions and defends against pathogens, its dysbiosis has been increasingly associated with oral diseases such as periodontitis and dental caries, as well as systemic conditions including cardiovascular disease and diabetes [2,3]. To characterize these microbial communities, researchers commonly employ diversity metrics: α-diversity measures the variety of species within a sample (richness and evenness), while β-diversity quantifies the dissimilarity in microbial community composition between samples [4,5].

Dental flossing is a fundamental oral hygiene behavior that can profoundly influence the oral microbiome by mechanically disrupting interproximal biofilms [6]. Epidemiologic evidence has increasingly linked regular flossing to improved long-term health outcomes, including a reduced risk of atherosclerotic cardiovascular disease and stroke [7,8]. Notably, a recent study utilizing the same NHANES dataset demonstrated a dose–response relationship between flossing frequency and lower cardiovascular disease prevalence, underscoring the public health relevance of this behavior within the U.S. population [7]. Controlled clinical trials have demonstrated that structured flossing interventions can alter microbial composition. For instance, these interventions can reduce specific periodontal pathogens [6] or improve clinical indicators such as gingival bleeding and plaque indices [9]. However, it remains unclear how routine flossing habits in the general population are associated with microbiome structure and diversity. This distinction is crucial from a public health perspective, as periodontal disease is highly prevalent, and flossing habits are highly variable, with a significant proportion of adults demonstrating inconsistent or minimal adherence [10].

Furthermore, whether the relationship between flossing and the microbiome is consistent across different segments of the population is poorly understood. Factors such as smoking, age, and socioeconomic status are well-established contributors to oral microbiome [11,12]. Emerging research suggests that oral health behaviors themselves, including flossing, are strongly correlated with oral health literacy and socioeconomic factors [13]. It is plausible that the effect of flossing may be attenuated in groups with established dysbiosis or amplified in groups where oral hygiene practices may have a greater relative impact. Systematic assessment of such effect modification is limited but is essential for informing targeted and effective oral health recommendations.

To address these gaps, this study utilized data from the National Health and Nutrition Examination Survey (NHANES) 2009–2012. We hypothesized that higher flossing frequency would be associated with reduced oral microbiome α-diversity and altered β-diversity. The null hypothesis is that there is no significant association between dental flossing frequency and oral microbiome α-diversity and altered β-diversity after accounting for confounders. The specific aims of this study were to:

Aim1: Examine the association between dental flossing frequency and oral microbiome α- and β-diversity.

Aim2: Explore whether the associations between flossing frequency and oral microbiome α-diversity differ across subgroups defined by smoking status, age, and socioeconomic factors.

## Methods

### Study design and population

The NHANES, conducted by the National Center for Health Statistics (NCHS), is an ongoing cross-sectional survey designed to assess the health and nutritional status of the noninstitutionalized civilian population in the U.S. [10]. The survey employed a complex, multistage probability sampling design to collect data biennially. NCHS survey weights were applied to account for differential selection probabilities and nonresponse, thereby enabling the generation of nationally representative estimates. Informed consent was obtained from all participants after the survey protocol was approved by the NCHS Research Ethics Review Board. No additional institutional review board approval was required for this secondary public-available data analysis.

### NHANES oral microbiome 2009–2012 sub-study

A subset of NHANES 2009–2012 participants aged 14–69 years was invited to mobile examination sites to participate in the Oral Microbiome sub-study, which aimed to characterize oral microbial profiles in a representative sample of U.S. adults [14]. Inclusion in this sub-study required completion of both the household interview and Mobile Examination Center (MEC) visit, as well as provision of oral rinse samples. Physical examinations were performed, and biological samples—including oral rinse samples for microbiome analysis—were collected. This study utilized publicly available data from the 2009–2010 and 2011–2012 survey cycles, with unweighted examination response rates of 77.3% and 69.5%, respectively [15].

### Analytic sample

**Figure 1.**
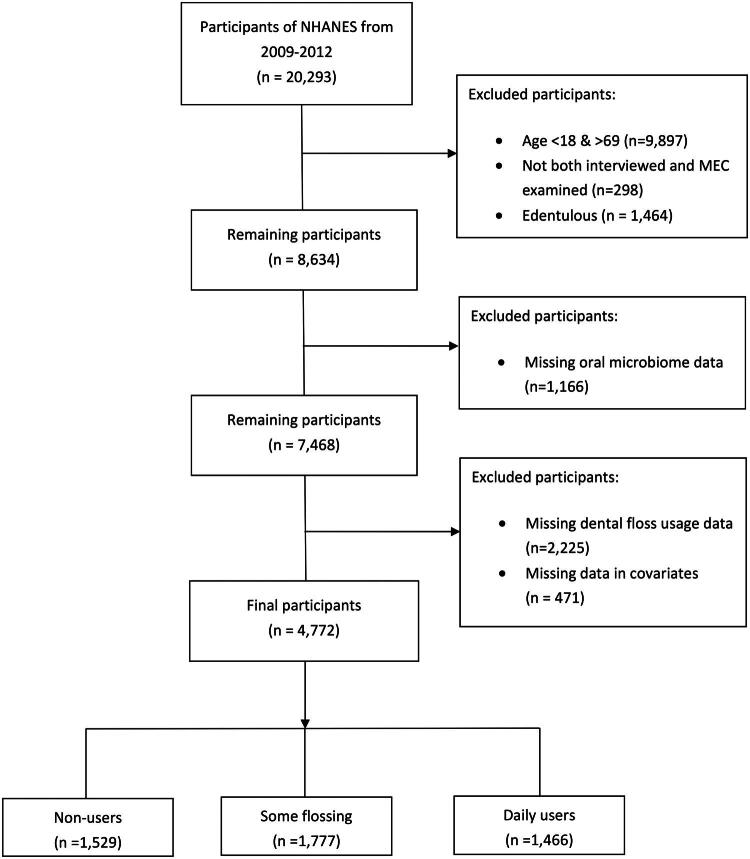
Sample creation flowchart (National Health and Nutrition Examination Survey, 2009–2012).

For this analysis, participants were drawn from the oral microbiome sub-study. Initial exclusions included participants aged <18 or >69 years, those who did not complete both the household interview and MEC examination, and those who were edentulous. Participants with missing oral microbiome data were then excluded. Because dental floss usage data were only collected for participants aged ≥30 years, participants younger than 30 were naturally excluded at this step. Finally, participants with missing data on prespecified covariates were excluded. The final analytic sample included participants aged 30–69 years who were dentate and had complete data on oral microbiome measures, dental floss usage, and prespecified covariates. The sample selection process is summarized in Figure 1.

## Measures

Oral Microbiome: Oral rinse samples were collected by trained examiners following standardized protocols. Participants rinsed with a mouthwash for 5 s and then gargled three times for 5 s, and the solution was then collected for downstream analysis. DNA was extracted from these samples and subjected to 16S rRNA gene sequencing targeting the V4 hypervariable region. Sequencing was performed on an Illumina HiSeq 2500 platform (2 × 125 bp, Illumina Inc., San Diego, CA, USA). Amplicon sequence variants (ASVs) were generated using a bioinformatic pipeline where initial processing utilized QIIME 1, and ASVs were inferred from the forward reads using the DADA2 algorithm (following the NHANES pipeline) run within R, allowing detailed characterization of oral microbial communities, consistent with procedures described in the NHANES laboratory protocol [16] and previous analyses [14].

To quantify the diversity of oral microbial communities, four α-diversity measures were calculated: Observed ASVs (representing species richness), Shannon–Weiner Index (capturing both richness and evenness), Inverse Simpson Index (emphasizing dominant species diversity), and Faith’s Phylogenetic Diversity (reflecting phylogenetic breadth). These indices collectively characterize within-sample microbial diversity and served as the primary outcome variables in subsequent analyses.

Dental Flossing Behavior: The exposure was self-reported dental flossing [17] behavior, categorized as ‘non-user’ (0 days in the last week), ‘some flossing’ (1–6 days/week), or ‘daily user’ (7 days/week) [18] based on the response to the question: “In the last seven days, how many days did you use dental floss or any other dental cleaning device?”

Covariates: Based on prior literature [19–21], we included covariates in the analysis to account for potential confounding factors affecting oral microbial composition. These included age (categorized as 30–39, 40–49, 50–59, and 60–69 years), sex (female or male), race/ethnicity (Mexican American, non-Hispanic Black, non-Hispanic White, Other Hispanic, and Other Race including multi-racial), educational attainment (less than high school, high school graduate/GED or equivalent, and above high school), marital status (married/living with partner, never married, divorced/widowed/separated), and the family income-to-poverty ratio (PIR). The PIR represents the family income divided by the poverty threshold for the survey year, which varies according to family size and geographic location. Income status was categorized as <1 (below poverty level), 1–1.999 (low income), 2–2.999 (moderate income), and ≥3 (higher income). Health behaviors and conditions included smoking status (categorized as current, former, or never smokers), self-reported histories of diabetes and hypertension, and clinically assessed periodontal disease [22] status (classified as none, mild, moderate, or severe).

### Sample size consideration

This study is a secondary analysis of NHANES 2009–2012 Oral microbiome sub-study; therefore, no a prior sample size calculation was performed, as the available sample size was determined by the NHANES complex, multistage probability sampling design and data availability. After applying prespecified inclusion and exclusion criteria (Figure 1), the final analytic sample included 4,772 participants, allowing sufficiently precise estimation of association between flossing frequency and oral microbiome diversity measures.

### Statistical analysis

To account for differential selection probabilities and nonresponse, analyses utilized MEC examination weights, which are critical for oral microbiome sub-study analyses [23]. Descriptive statistics are presented as weighted means ± SD for continuous variables (compared using design-adjusted *t* tests) and as unweighted counts with weighted percentages for categorical variables (compared using Rao–Scott χ^2^ tests).

We used survey-weighted univariate and multivariate linear regression models to examine the associations between each α-diversity measure described above and dental floss usage (non-user, some flossing, daily user). Model 1 was first constructed for each association without any covariate adjustment, using non-users as the reference group. Model 2 adjusted for age, race, gender, income-to-poverty ratio, marital status, and education. Model 3 further adjusted for smoking status, diabetes, hypertension, and periodontal disease. Given the established impact of smoking on the oral microbiome and its influence on oral hygiene and periodontal health, α-diversity subgroup analyses stratified by smoking status (never, former, current smokers) were also conducted [24,25].

We assessed β-diversity using Bray–Curtis dissimilarity [26], unweighted UniFrac distance, and weighted UniFrac distance. Principal coordinate analysis (PCoA) was performed using the cmdscale function to visualize the distribution of oral microbiome communities by flossing frequency (non-user, some flossing, daily-user). The proportion of variance (*R*^2^) in β-diversity explained by flossing frequency was evaluated using both unadjusted and fully adjusted models *via* PERMANOVA. Adjusted models controlled for age, race, gender, income-to-poverty ratio, marital status, education, smoking status, diabetes, hypertension, and periodontal disease. Pairwise PERMANOVA comparisons between flossing frequency groups were also performed, with *p* values adjusted using the Bonferroni correction.

All statistical tests were two-sided with statistical significance defined as *p* < 0.05. Normality was assessed using skewness, with values between ±2 considered sufficiently normal for analyses [27]. All analyses were performed in R version 4.4.2 (RStudio, Boston, Massachusetts). Survey-weighted regression analyses were conducted using the survey package (v4.4-2). Microbiome β-diversity calculations-including PCoA and PERMANOVA-were performed using the vegan (v2.7-1).

## Results

### Sample characteristics

For the 4,772 participants in the analytical sample, we classified 1,529 (32.0%) as non-users of dental floss, 1,777 (37.2%) as some flossing (1–6 days/week), and 1,466 (30.7%) as daily users. Overall, about one-third reported daily flossing, with the prevalence highest among women (59% vs. 37% for non-users) and those with higher socioeconomic status (14% vs. 24% with less than high school education for daily users vs. non-users; 11% vs. 19% with income-to-poverty ratio <1). Further sociodemographic and health-related characteristics across flossing groups are presented in Table 1.

**Table 1.** Characteristics of participants by dental floss usage groups (NHANES, 2009-2012, *N* = 4,772).^a^

	Total		Dental floss usage						
			Non-user		Some flossing		Daily user		
	*N* = 4,772		*N* = 1,529		*N* = 1,777		*N* = 1,466		*p* values
	*N*	%	*N*	%	*N*	%	*N*	%	
Age groups (years)									0.007
30–39	1,353	28	431	30	584	30	338	23	
40–49	1,313	29	408	30	508	30	397	28	
50–59	1,116	26	356	25	386	25	374	28	
60–69	990	17	334	15	299	15	357	21	
Age (years), Mean (SD)		47.46 (10.70)		46.71 (10.66)		46.85 (10.65)		49.00 (10.66)	<0.001
Sex									<0.001
Female	2,379	50	598	37	909	52	872	59	
Male	2,393	50	931	63	868	48	594	41	
Race and ethnicity									<0.001
Mexican American	771	8.5	305	11	240	7	226	9	
Non-Hispanic Black	1,083	11	375	13	381	10	327	11	
Non-Hispanic White	1,889	68	550	62	813	73	526	65	
Other Hispanic	491	5.5	143	6	149	4	199	7.2	
Other race – including multi-racial	538	7	156	8	194	6	188	7.8	
Education									<0.001
Less than high school	1,131	15	546	24	292	10	293	14	
High school graduate/GED or equivalent	1,004	20	376	24	337	18	291	19	
Above high school	2,637	65	607	52	1,148	72	882	67	
Marital status									0.734
Married/living with partner	3,151	71	976	69	1,213	72	962	71	
Never married	599	11	201	12	216	11	182	11	
Divorced/widowed/separated	1,022	18	352	19	348	18	322	18	
Income-to-poverty ratio									<0.001
<1 (below poverty level)	992	13	442	19	298	10	252	11	
1–1.999	1,227	18	480	24	397	15	350	17	
2–2.999	614	14	192	15	244	14	178	12	
≥3	1,939	55	415	42	838	61	686	60	
Smoking status									<0.001
Current smokers	1,030	19	428	26	351	18	251	16	
Former smokers	1,077	25	319	20	400	26	358	27	
Never smokers	2,665	56	782	54	1,026	56	857	58	
Self-reported diabetes									0.052
No	4,263	92	1,344	91	1,592	92	1,327	94	
Yes	509	8	185	9	185	8	139	6	
Self-reported hypertension									0.070
No	3,217	70	995	67	1,232	72	990	72	
Yes	1,555	30	534	33	545	28	476	28	
Periodontal									
None	2,332	58	556	46	980	62	796	63	<0.001
Mild	326	6.5	121	8	136	7	69	4	
Moderate	1,524	27	558	32	501	25	465	27	
Severe	590	8.5	294	14	160	6	136	6	

*Note*: SD, standard deviation.

^a^ Categorical variables are presented as unweighted counts (*N*) and weighted percentages. Continuous variables are presented as weighted means and standard deviations. *p* values were derived from weighted analyses (Rao–Scott χ² or design-adjusted *t* test) to account for NHANES’ complex survey design and ensure nationally representative inferences.

### α-diversity

Survey-weighted regression indicated a dose-response relationship: daily floss users had the lowest Faith’s Phylogenetic Diversity and fewest Observed ASVs compared to non-users, while some flossing user showed intermediate values. For Faith’s PD, the association remained significant for daily users across all models, with coefficients ranging from −1.57 (95% CI: −1.86 to −1.28, *p* < 0.001) in the unadjusted model to −0.88 (95% CI: −1.20 to −0.56, *p* < 0.001) in the fully adjusted model. For some flossing, the association was significant in the unadjusted model (β = −0.80, 95% CI: −1.12 to −0.55, *p* < 0.001) and Model 2 (β = −0.33, 95% CI: −0.60 to −0.05, *p* = 0.024) but became non-significant after full adjustment (β = −0.21, 95% CI: −0.48 to 0.06, *p* = 0.113), generally showing a trend toward reduced diversity. Similarly, Observed ASVs were consistently lower among daily users, with coefficients from −19.74 (95% CI: −23.56 to −15.93, *p* < 0.001) in the unadjusted model to −11.46 (95% CI: −15.62 to −7.29, *p* < 0.001) after full adjustment. Some flossing showed a significant reduction in the unadjusted model (β = −10.61, 95% CI: −14.38 to −6.84, *p* < 0.001) and Model 2 (β = −4.38, 95% CI: −8.01 to −0.75, *p* = 0.021) although the association became non-significant after fully adjustment (β = −2.97, 95% CI: −6.55 to 0.60, *p* = 0.095), suggesting a trend toward reduced diversity. For the Shannon–Weiner index, daily users showed a significant reduction in the unadjusted model (β = −0.20, 95% CI: −0.28 to −0.13, *p* < 0.001) and after full adjustment (β = −0.10, 95% CI: −0.19 to −0.02, *p* = 0.019). Some flossing showed a significant difference only in the unadjusted model (β = −0.10, 95% CI: −0.16 to −0.03, *p* = 0.005) but not after adjustment. No significant differences were found for the Inverse Simpson index in any model for either some flossing or daily users compared to non-users (Table 2).

**Table 2.** Survey-weighted associations between dental floss usage and α-diversity measures (*n* = 4,772).^a^

	Model 1			Model 2			Model 3		
	Β	95% CI	*p* values	β	95% CI	*p* values	β	95% CI	*p* values
**Faith phylogenetic diversity**									
Non-user	*Ref*								
Some flossing	−0.80	(–1.12, −0.55)	**<0.001**	−0.33	(–0.60, −0.05)	**0.024**	−0.21	(–0.48, 0.06)	0.113
Daily user	−1.57	(–1.86, −1.28)	**<0.001**	−1.04	(–1.36, −0.73)	**<0.001**	−0.88	(–1.20, −0.56)	**<0.001**
**Simpson index**									
Non-user	*Ref*								
Some flossing	*0.00*	*(*–*0.01, 0.00)*	*0.307*	0.00	(–0.01, 0.01)	0.653	0.00	*(0.00, 0.01)*	*0.546*
Daily user	*0.00*	*(*–*0.01, 0.00)*	*0.122*	0.00	(−0.01, 0.01)	0.798	0.00	(–0.01, 0.01)	0.936
Observed ASVs									
Non-user	*Ref*								
Some flossing	−10.61	(–14.38, −6.84)	**<0.001**	−4.38	(–8.01, −0.75)	**0.021**	*−2.97*	*(*–*6.55, 0.60)*	*0.095*
Daily user	−19.74	(–23.56, −15.93)	**<0.001**	−13.43	(−17.50, −9.36)	**<0.001**	−11.46	(–15.62, −7.29)	**<0.001**
**Shannon–Weiner index**									
No	*Ref*								
Some	−0.10	(–0.16, −0.03)	0.005	−0.02	(–0.08, 0.05)	0.635	*0.00*	*(*–*0.07, 0.07)*	*0.978*
Daily	−0.20	(–0.28, −0.13)	**<0.001**	−0.12	(–0.20, −0.04)	**0.005**	−0.10	(–0.19, −0.02)	**0.019**

CI, Confidence interval; Ref: Reference. ^a^Survey-weighted linear regression analysis was conducted. Model 1 presents the bivariate association between dental floss usage and four α-diversity measures, using the non-user group as the reference. Model 2 was adjusted for age, race, gender, income-to-poverty ratio, marital status, and education. Model 3 was further adjusted for smoking status, diabetes, hypertension, and periodontal disease.

Subgroup analyses revealed that the associations between daily flossing and lower Faith’s PD and Observed ASVs were most pronounced among younger participants (30–39 and 40–49 years), those with incomes below the poverty level, and never or former smokers. The associations for some flossing were generally weaker or but showed a trend across subgroups. No clear associations were observed in older age groups, higher-income groups, or current smokers (Table 3).

**Table 3.** Survey-weighted associations between dental floss usage and α-diversity, stratified by age, sex, SES, and smoking status.^a^

		Faith phylogenetic diversity			Simpson index			Observed ASVs			Shannon–Weiner index		
		β	95% CI	*p* values	β	95% CI	*p* values	β	95% CI	*p* values	β	95% CI	*p* values
**Age groups (years)**													
** 30–39**													
	Some	−0.47	(–1.02, 0.09)	0.093	0.00	(–0.01, 0.02)	0.502	−6.83	(–13.87, 0.20)	0.056	0.00	(–0.13, 0.13)	0.986
	Daily	−1.04	(–1.75, −0.32)	0.009	0.01	(–0.01, 0.02)	0.404	−14.22	(–23.80, −4.65)	0.007	−0.06	(–0.20, 0.08)	0.358
** 40**–**49**													
	Some	−0.30	(–0.88, 0.29)	0.296	0.00	(–0.01, 0.01)	0.467	−4.86	(–12.66, 2.94)	0.200	−0.06	(–0.16, 0.03)	0.163
	Daily	−1.07	(–1.72, −0.41)	0.004	−0.01	(–0.02, 0.01)	0.377	−14.24	(–22.55, −5.92)	0.003	−0.16	(–0.28, −0.04)	0.013
** 50**–**59**													
	Some	−0.02	(–0.55, 0.51)	0.940	0.01	(–0.01, 0.02)	0.273	−1.03	(–8.10, 6.04)	0.757	0.06	(–0.07, 0.19)	0.325
	Daily	−0.57	(–1.26, 0.12)	0.097	0.01	(–0.01, 0.02)	0.464	−8.40	(–16.97, 0.16)	0.054	−0.06	(–0.23, 0.10)	0.408
** 60**–**69**													
	Some	−0.04	(–0.78, 0.69)	0.896	0.00	(–0.02, 0.02)	0.911	2.71	(–6.54, 11.96)	0.532	0.01	(–0.24, 0.26)	0.953
	Daily	−1.10	(–1.89, −0.31)	0.011	−0.01	(–0.02, 0.00)	0.171	−10.64	(–21.23, −0.06)	0.049	−0.17	(–0.36, 0.03)	0.089
**Sex**													
** Female**													
	Some	−0.31	(–0.76, 0.15)	0.166	0.01	(–0.01, 0.02)	0.387	−4.05	(–9.99, 1.88)	0.161	0.02	(–0.09, 0.14)	0.653
	Daily	−0.93	(–1.49, −0.36)	0.004	0.00	(–0.01, 0.02)	0.504	−11.57	(–18.62, −4.52)	0.004	−0.07	(–0.20, 0.06)	0.268
** Male**													
	Some	−0.159	(–0.52, 0.20)	0.357	0.000	(–0.01, 0.01)	0.893	−2.316	(–7.01, 2.37)	0.303	−0.023	(–0.09, 0.04)	0.439
	Daily	−0.891	(–1.21, −0.57)	<0.001	−0.005	(–0.01, 0.00)	0.264	−12.05	(–16.15, −7.94)	<0.001	−0.144	(–0.23, −0.06)	0.003
**Income-to-poverty ratio**													
** <1 (below poverty level)**													
	Some	−0.80	(–1.37, −0.24)	0.009	0.00	(–0.01, 0.01)	0.758	−11.49	(–18.89, −4.09)	0.005	−0.09	(–0.22, 0.04)	0.155
	Daily	−0.83	(–1.43, −0.23)	0.010	0.00	(–0.02, 0.01)	0.563	−11.82	(–20.29, −3.35)	0.010	−0.16	(–0.30, −0.01)	0.036
** 1**–**1.999**													
	Some	0.04	(–0.53, 0.60)	0.893	0.001	(–0.01, 0.01)	0.846	−1.22	(–8.29, 5.86)	0.716	−0.01	(–0.11, 0.09)	0.837
	Daily	−0.61	(–1.30, 0.08)	0.077	0.00	(–0.01, 0.01)	0.974	−9.23	(–18.13, −0.33)	0.043	−0.08	(–0.22, 0.05)	0.205
** 2**–**2.999**													
	Some	−0.57	(–1.51, 0.37)	0.216	0.00	(–0.02, 0.01)	0.582	−5.98	(–16.60, 4.65)	0.246	−0.08	(–0.22, 0.06)	0.248
	Daily	−1.41	(–2.27, −0.55)	0.004	0.00	(–0.02, 0.01)	0.685	−17.33	(–28.52, −6.14)	0.005	−0.16	(–0.33, 0.02)	0.080
** ≥3**													
	Some	−0.01	(–0.42, 0.40)	0.954	0.01	(–0.01, 0.02)	0.339	−0.01	(–4.95, 4.93)	0.996	0.06	(–0.04, 0.17)	0.208
	Daily	−0.83	(–1.25, −0.41)	0.001	0.00	(–0.01, 0.01)	0.841	−10.53	(–15.76, −5.30)	0.001	−0.08	(–0.18, 0.03)	0.148
**Smoking status**													
**Never smoker**													
	Some	−0.43	(–0.79, −0.07)	0.024	0.00	(–0.01, 0.01)	0.542	−6.29	(–11.03, −1.55)	0.013	−0.06	(–0.14, 0.02)	0.111
	Daily	−1.02	(–1.46, −0.58)	<0.001	0.00	(–0.01, 0.01)	0.662	−13.40	(–18.88, −7.93)	<0.001	−0.12	(–0.24, 0.00)	0.050
**Former smoker**													
	Some	−0.24	(–0.92, 0.45)	0.464	0.00	(–0.01, 0.02)	0.503	−3.28	(–11.16, 4.59)	0.382	0.03	(–0.10, 0.17)	0.604
	Daily	−1.01	(–1.62, −0.40)	0.003	0.00	(–0.01, 0.01)	0.735	−14.00	(–21.88, −6.13)	0.002	−0.14	(–0.26, −0.02)	0.025
**Current smoker**													
	Some	0.29	(–0.30, 0.89)	0.307	0.01	(–0.01. 0.03)	0.230	4.75	(–2.81, 12.31)	0.198	0.10	(–0.06, 0.26)	0.206
	Daily	−0.47	(–1.10, 0.16)	0.131	0.00	(–0.01, 0.02)	0.516	−5.60	(–13.51, 2.31)	0.150	−0.06	(–0.20, 0.09)	0.431

CI, Confidence interval; Demo, Demographic; SES, Social economic status.

^a^Survey-weighted linear regression analysis was conducted, using the non-user group as the reference. Models were adjusted for age, race, gender, income-to-poverty ratio, marital status, education, smoking status, periodontal disease, diabetes, and hypertension.

### β-diversity

**Figure 2.**
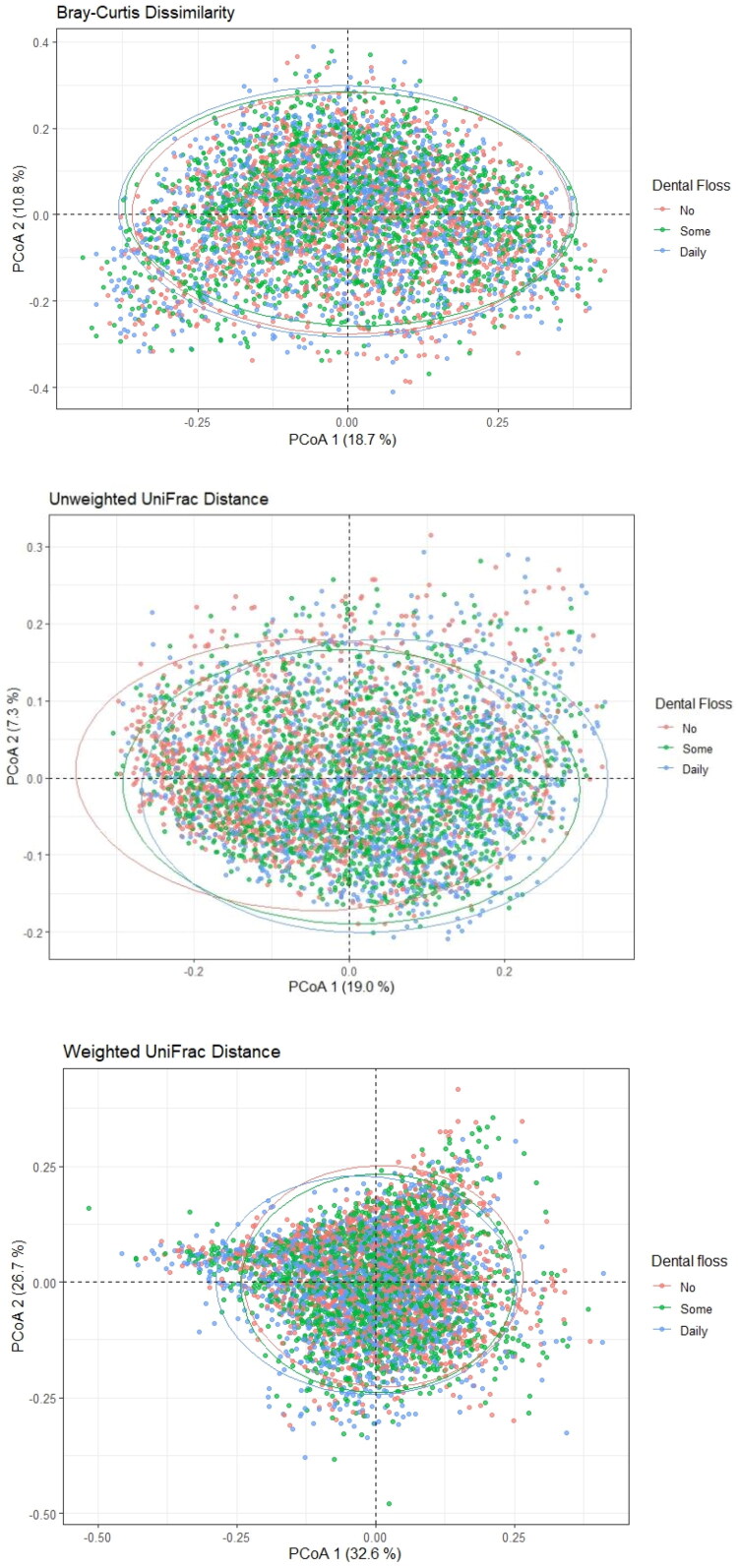
Principal coordinate analysis (PCoA) of oral microbiome β-diversity, stratified by dental floss usage (non-user, some flossing, daily user). Plots are based on (A) Bray–Curtis dissimilarity, (B) unweighted UniFrac distance, and (C) weighted UniFrac distance (NHANES 2009–2012; *N* = 4,772). Ellipses represent 95% confidence intervals for each group. Axes indicate the proportion of variance explained by the principal coordinates.

PCoA plots based on Bray–Curtis, unweighted UniFrac, and weighted UniFrac distances showed considerable overlap among the three flossing frequency groups, though centroid shifts suggested subtle compositional differences (Figure 2).

Despite the high degree of overlap, PERMANOVA confirmed statistically significant differences in community composition among flossing frequency groups for all β-diversity metrics. For Bray–Curtis dissimilarity, the unadjusted model explained 0.004% of variance (*p* < 0.001), increasing to 0.059% after adjustment (*p* < 0.001). For unweighted UniFrac, the unadjusted and adjusted models explained 0.009% and 0.090% of variance, respectively (both *p* < 0.001). For weighted UniFrac, the unadjusted model explained 0.007% of variance, rising to 0.066% after adjustment (*p* < 0.001) (Table 4). Pairwise PERMANOVA comparisons indicated significant differences in microbial community composition across all flossing groups (non-users vs. some, non-users vs. daily, some vs. daily) after Bonferroni correction, consistent across Bray–Curtis, unweighted UniFrac, and weighted UniFrac metrics (Supplementary Tables 1–3). Although effect sizes were small, daily users consistently showed the greatest separation from non-users across all three distance measures.

**Table 4.** PERMANOVA results for β-diversity comparing dental floss usage groups (*N* = 4,772).

	*p* value (*R*^2^, %)	
β-diversity	No adjustment	Full adjustment^a^
Bray–Curtis dissimilarity	*p* = 0.001 (0.004%)	*p* = 0.001 (0.059%)
Unweighted UniFrac distance	*p* = 0.001 (0.009%)	*p* = 0.001 (0.090%)
Weighted UniFrac distance	*p* = 0.001 (0.007%)	*p* = 0.001 (0.066%)

PERMANOVA: permutational multivariate analysis of variance. ^a^Models were adjusted for age, race, gender, income-to-poverty ratio, marital status, education, diabetes, hypertension, and periodontal disease.

## Discussion

This large epidemiological study found a dose-dependent association where increasing frequency of dental flossing, particularly daily use, was associated with reduced richness and phylogenetic diversity of the oral microbiome, as well as subtle but significant shifts in community structure. We therefore reject the null hypothesis of no association between dental flossing frequency and oral microbiome α-diversity and altered β-diversity. The association was most pronounced in younger adults, those with lower socioeconomic status, and never or former smokers, but absent among current smokers. For intermediate flossing frequency (1–6 days/week), associations showed a trend toward reduced diversity but were not statistically significant after full adjustment.

The observed dose–response relationship, with daily flossing showing the strongest inverse association with richness and phylogenetic diversity, aligns with findings from controlled trials and large-scale population studies. For example, Sreenivasan et al. reported that an intensive regimen of antiseptic mouth rinse and flossing reduced Shannon diversity in supragingival plaque [5,28]. Our study extends this evidence to a broad, community-dwelling population, suggesting that more frequent routine flossing is associated with measurable reductions in the number and phylogenetic range of oral microbial taxa. This supports the mechanistic hypothesis that physical disruption of interproximal biofilms removes bacterial biomass, limiting niche availability and reducing diversity [29]. The persistence of this association after adjusting for periodontal disease status suggests that the link is not merely a proxy for underlying oral health. This reduction in diversity may reflect a shift toward a health-associated biofilm, as some studies indicate that higher diversity can sometimes signal dysbiosis in periodontal disease [30]. This interpretation is supported by a recent population-based study which found that better oral hygiene (including flossing and mouthwash use) was linked to lower gingival fluid bacterial diversity, while self-reported frequent bleeding was associated with higher diversity and enrichment of periodontal pathogens like Porphyromonas and Treponema [9]. This suggests that in the context of hygiene, reduced diversity may signify beneficial removal of pathogenic biomass rather than ecosystem impoverishment.

Our findings are validated by the complex and seemingly paradoxical relationship between oral microbiome diversity and health outcomes. While increased diversity often signifies ecosystem stability and health in many body sites, the oral cavity presents a unique case: higher α-diversity has been associated with both reduced all-cause mortality [31,32] and increased periodontal disease [33]. This implies that not all diversity is beneficial; the compositional and functional characteristics of the community are crucial. In our study, the observed dose-dependent reduction in diversity with increasing flossing frequency may thus reflect the removal of disease-associated taxa and a shift toward a more stable, health-associated community, rather than a loss of beneficial species. This view is supported by studies showing that improved oral hygiene reduces the abundance of periodontal pathogens such as *Porphyromonas gingivalis* and *Treponema denticola* [34], and the complex relationship is further highlighted by recent NHANES-based analyses which have linked higher α-diversity to a reduced risk of all-cause and cardiovascular mortality [35], a finding that appears paradoxical in the context of periodontal disease but emphasizes the context-dependency of diversity metrics. Emerging evidence suggests that mechanical interventions like flossing may influence local biochemical milieus beyond simple biofilm removal. For instance, orthodontic tooth movement, a controlled mechanical force, has been shown to induce oxidative stress and alter the expression of microRNAs (miRNAs) in gingival crevicular fluid, which in turn regulate bone remodeling and inflammation [36]. It is plausible that routine flossing, as a repeated mild mechanical stimulus, could impart subtle, localized biochemical changes that select for or against certain microbial taxa, contributing to the observed β-diversity shifts. This paradox highlights the need to interpret microbial diversity metrics within specific ecological and clinical contexts [37,38]. Clinically, it suggests that reduced diversity with daily flossing may not indicate harm, but rather a targeted removal of pathogenic taxa, emphasizing the importance of considering both composition and function when evaluating oral microbiome health.

Subgroup analyses of α-diversity revealed important effect modifications. Associations were strongest for daily flossing in younger adults, those with lower income, and never or former smokers. For some flossing users, trend-level reductions in Observed ASVs and Faith’s PD were observed, though these did not reach statistical significance, consistent with β-values that were attenuated. The attenuated associations in older adults and higher-income groups may reflect cumulative lifestyle, health, or medication influences that outweigh the effect of flossing. The most notable effect modifier was smoking status. The absence of a significant association between daily flossing and α-diversity in current smokers suggests that the profound dysbiotic effects of smoking may overwhelm the more subtle influence of flossing [39]. Cigarette smoke exposure creates a hypoxic oral environment, depleting oxygen-utilizing taxa (e.g. Neisseria, Haemophilus) and enriching anaerobic pathogens (e.g. Porphyromonas, Prevotella, Treponema) [39]. This radical restructuring of the microbial ecosystem likely creates a state in which mechanical biofilm removal *via* flossing is insufficient to alter overall diversity metrics within already compromised communities [40]. This finding underscores the necessity of comprehensive management for high-risk patients. It aligns with recent evidence emphasizing that effective long-term periodontal health, especially in challenging cases, depends on professionally delivered supportive periodontal care that includes subgingival instrumentation, coupled with patient adherence and lifestyle modifications like smoking cessation [41]. Clinically, this indicates that flossing should be combined with broader oral health strategies, such as smoking cessation and professional preventive care, in high-risk populations.

Our results advocate for a “precision oral health” approach, where recommendations are tailored to individual risk profiles. For the general population and lower-risk groups, promoting daily flossing remains a simple, low-cost strategy to promote a healthier microbial ecology. For high-risk individuals, such as current smokers or those with established periodontitis, a more intensive regimen is warranted. This could include adjunctive chemotherapeutic agents. For example, the adjunctive use of an essential oil mouth rinse has been shown to provide significant reductions in plaque and gingivitis beyond mechanical cleaning alone [42]. Future research should explore the synergistic effects of combining mechanical cleaning with bioactive agents. Interestingly, remineralizing agents like zinc hydroxyapatite toothpaste have proven effective in improving periodontal indices and sensitivity in vulnerable populations like children with asthma [43], while novel biomimetic varnishes containing theobromine show promising remineralization potential [44]. Investigating whether such agents, combined with optimal flossing, can further optimize the oral microbiome and clinical outcomes represents a fertile ground for future interdisciplinary research.

## Clinical implications

Overall, these findings suggest that daily flossing is a simple, low-cost, and scalable behavior that promotes healthier oral microbial communities and may reduce the risk of periodontal disease. Effects were strongest among younger adults and socioeconomically disadvantaged groups, highlighting the public health value of emphasizing routine flossing in these populations. Conversely, the lack of effect in current smokers suggests that flossing alone is insufficient in high-risk populations, who may require combined strategies including smoking cessation and professional dental care, consistent with the principles of effective supportive periodontal therapy [41]. Trend-level associations for some flossing users suggest modest benefits, reinforcing the superiority of daily flossing. Beyond oral health, maintaining a balanced oral microbiome through daily flossing could also help reduce systemic inflammation and potentially lower the risk of cardiovascular disease, diabetes, and cognitive decline, consistent with emerging evidence linking oral microbiota to systemic outcomes. These results support public health messaging promoting daily flossing while encouraging precision oral health strategies tailored to individual risk profiles.

## Limitations

Several limitations must be acknowledged. First, the cross-sectional design of NHANES precludes causal inference or determination of temporal sequence. Second, oral hygiene behaviors were self-reported and subject to recall and social desirability biases. Third, despite extensive adjustment for potential confounders, residual confounding from unmeasured factors (e.g. detailed dietary habits, use of antimicrobial toothpaste, or genetic predisposition) may remain. Fourth, crucial data on toothbrushing frequency were not collected in the NHANES 2009-2012 cycles, preventing us from adjusting for this primary confounder. Although other behaviors like mouthwash use were examined, the relevant survey question pertained to therapeutic use rather than routine prevention. The lack of control for toothbrushing means that our observed associations may partially reflect differences in overall oral hygiene diligence rather than the independent association of flossing.

## Conclusion

This large epidemiological study demonstrates that increasing frequency of self-reported dental flossing, particularly daily use, is associated in a dose-dependent manner with reduced richness and phylogenetic diversity of the oral microbiome and discernible shifts in overall community structure. Trend-level reductions were observed for sub-daily flossing, though these did not reach statistical significance. Our findings help resolve the oral microbiome diversity–health paradox by suggesting that in the context of hygiene, reduced diversity may reflect a beneficial reduction in pathogenic species and biomass. The effect is modified by age, socioeconomic status, and smoking, underscoring the need for personalized oral health advice. Future research should prioritize longitudinal and interventional studies to establish causality and quantify dynamic microbial changes in response to different frequencies of flossing. Additionally, mechanistic studies are needed to elucidate how flossing alters key health- and disease-associated taxa and influences systemic health outcomes *via* pathways such as the oral–gut axis. Future studies should also be powered to examine potential effect modification in β-diversity across key subgroups such as smokers. Incorporating host-derived biomarkers alongside microbial profiling could enhance our ability to predict individual responses to oral hygiene interventions. By adopting these approaches, we can better inform personalized oral health recommendations and leverage the oral microbiome as a modifiable target for improving overall health.

## Supplementary Material

Supplementary table 3.docx

Supplementary table 1.docx

Supplementary table 2.docx

## Data Availability

The data used in this study are publicly available from the NHANES database (https://wwwn.cdc.gov/nchs/nhanes/omp/Default.aspx). Additional information or materials supporting the findings of this study are available from the corresponding author upon reasonable request.
