# Supplementary material for: Association between dental flossing frequency and oral microbiome in U.S. adults
Source: Ann Med. 2026 Jan 16;58(1):2614826. doi: 10.1080/07853890.2026.2614826 (PMC12818320; doi:10.1080/07853890.2026.2614826)
Supplement: Supplementary table 3.docx [file IANN_A_2614826_SM7637.docx]

| **Supplementary Table 3. Pairwise PERMANOVA Comparison of Weighted UniFrac Distance Across Dental Floss Usage Groups (N=4,772)** | | | | | |
| --- | --- | --- | --- | --- | --- |
|  |  |  | **Adjusted- p-Value ^a^ (R^2^, %)** | | |
| **Weighted UniFrac Distance** |  |  | **No adjustment** |  | **Full Adjustment ^b^** |
| Some flossing vs. Non-user |  |  | p=0.003 (0.003%) |  | P=0.003 (0.068%) |
| Some flossing vs. Daily user |  |  | p=0.003 (0.003%) |  | P=0.003 (0.065%) |
| Non-user vs. Daily user |  |  | P=0.003 (0.010%) |  | P=0.003 (0.068%) |
| Abbreviation: PERMANOVA: permutational multivariate analysis of variance.  ^a^ p-Values were adjusted for multiple comparisons using the Bonferroni correction (family-wise error rate control). ^b^ Models were adjusted for age, race, gender, income-to-poverty ratio, marital status, education, smoking status, diabetes, , hypertension, and periodontal disease. | | | | | |
